# Supplementary material for: Phytochemistry, Medicinal Properties, Bioactive Compounds, and Therapeutic Potential of the Genus Eremophila (Scrophulariaceae)
Source: Molecules. 2022 Nov 10;27(22):7734. doi: 10.3390/molecules27227734 (PMC9697388; doi:10.3390/molecules27227734)
Supplement: Supplementary file 1 [file molecules-27-07734-s001.zip › molecules-1998812-supplementary.pdf]

Supplementary Data

Review

Phytochemistry, medicinal properties, bioactive compounds and therapeutic potential of the genus *Eremophila* (Scrophulariaceae)

Ian Edwin Cock [1,*], Linn Baghtchedjian [2], Marie-Elisabeth Cordon [2] and Eléonore Dumont [2]

[1] Centre for Planetary Health and Food Security, Griffith University, Brisbane 4127, Australia

[2] Ecole De Biologie Industrielle, Cergy 95800, France

* Correspondence: i.cock@griffith.edu.au; Tel.: +61-737357637

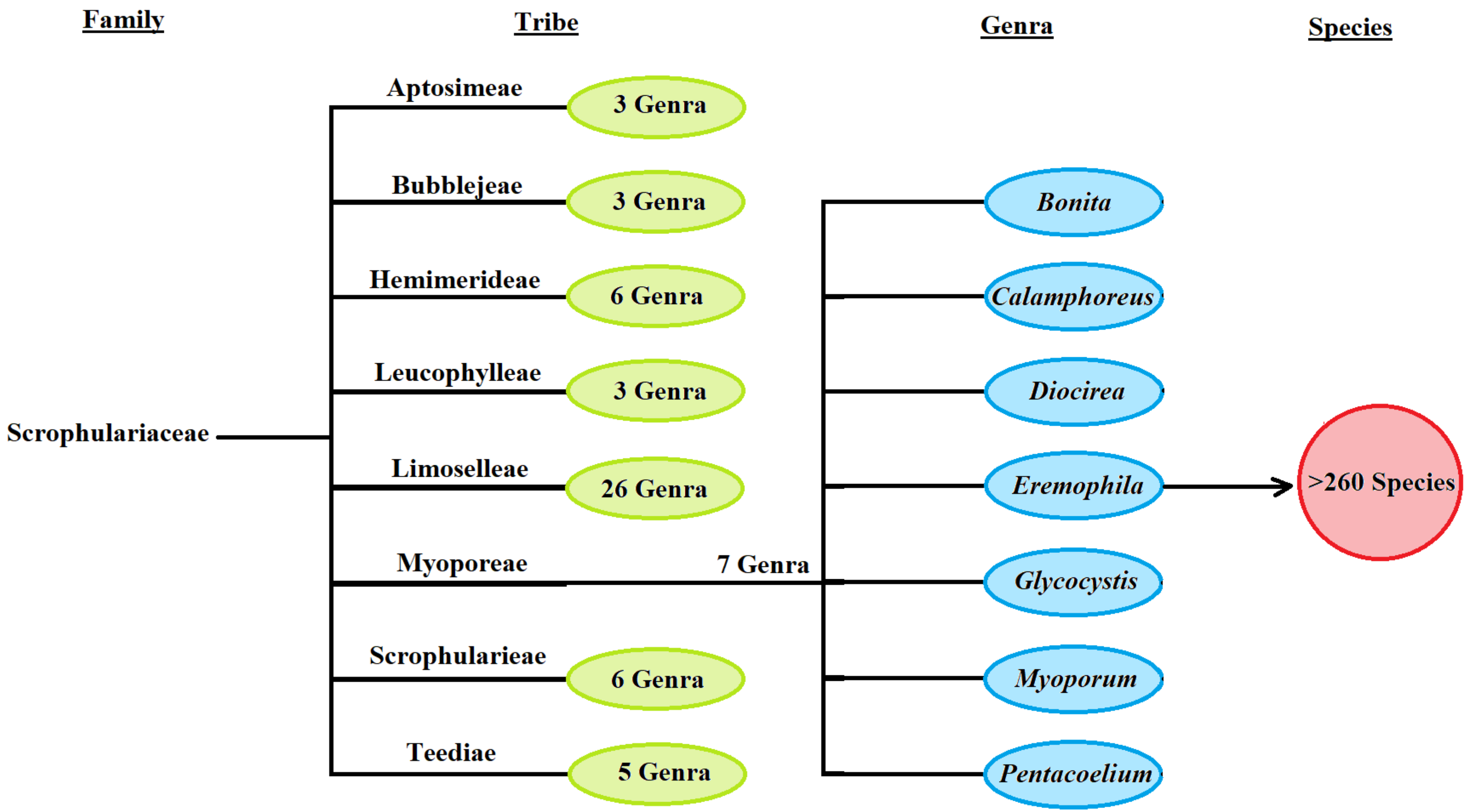

Figure S1. Classification of the genus *Eremophila* within the family Scrophulariaceae.

Table S1. The use of *Eremophila* spp. in traditional healing systems, including the indication, plant part used and how the medicine was traditionally prepared.

Species	Common/traditional name	Indication	Part used	Preparation method	References
Eremophila alternifolia R.Br.	Narrow-leaf fuchsia bush, emu bush, scented emu bush, round-leaf poverty bush, magenta emu bush, native honey suckles (English), Irmangka irmangka (Pitijantjatjara)	Sleeping aid and general well-being (tonic)	Leaves	Infusion, ingested	1,2
		Colds, influenza, coughs, headaches		Decoction, ingested	3
		Internal pain, analgesic, expectorant and decongestant		A paste is prepared by kneading of dry leaves with water. External application.	4,5,6,7
		Septic wounds			8
		Septic wounds		Body wash	3
Eremophila bignoniiflora (Benth.) F.Muell.	River angee, creek wilga, emu bush (English), Gooramurra, Kurubimi (Mudburra and Djingulu)	Laxative	Leaves	Decoction, ingested	6,8
		Purgative to treat extreme illness	Fruit	Decoction, ingested	8
		Cold and flu	Young leaves	Aqueous decoction is applied to the body.	4,5
		Headaches, colds, sinusitis, nasal congestion	Leaves and twigs	Preparation not specified. Applied by wrapping around the head.	4,5
Eremophila dalyana F.Muell.	Unknown	Relief of chest pains and colds.	Leaves	Combined with animal fat to produce a body rub, which is applied directly.	9
		Body wash for scabies.		Decoction is prepared and used as a body wash.	10

Eremophila duttonii F.Muell.	Kangaroo bush, red poverty bush (English), Arreh Indenjlie or Agherre intenthe (Aranda), Muntjunpa (Pitijantjatjara), Munyunpa (Yankunytjatjara)	Sores, cuts, colds, influenza, eye and ear complaints, minor dermal wounds, and infected lesions. Has insect repellant properties (especially for scabies mites).	Leaves	Antiseptic wash	1,3
		Sore throats and other respiratory tract infections.	Leaves	Decoction is ingested.	4,5
Eremophila elderi F.Muell.	Unknown	Colds and respiratory illnesses.	Leaves	A decoction is prepared and applied externally.	10
Eremophila fraseri F.Muell.	Turpentine bush, turpentine plant, wax bush (English),	Alleviate cold symptoms.	Leaves	Decoction. Application unspecified.	11
		Relief of toothache and rheumatism.		Unspecified.	4,5
Eremophila freelingii F.Muell.	Limestone fuchsia, rock fushia bush (English), Ruatta, Arrethe (Aranda), Aratja (Pitjantjatjara and Yankunytjatjara), Miyinypa (Warlpiri)	Headaches, fever, and chest pain.	Leaves	Inhalation of steam from a hot bath containing leaves.	4,5,12
		Antiseptic wash for open sores, scabies, and infected cuts.		Bath made from decoction.	4,5,3
		Colds and cough.		Infusion is ingested.	13
		To treat diarrhea		Decoction, ingested.	4,5,14
		Used as a pillow to promote rest and for sick head.		Used externally.	4,5,15
		General well-being.		Infusion is consumed.	9
		Perforation of nasal septum.	Twig	External usage.	4,5,16
Eremophila gilesii F.Muell.	Unknown	Headaches and chest pain.	Leaves	Decoction, application unspecified.	12
		Antibacterial agent to treat sores.		Body wash	5,17
		Colds		Infusion is consumed	9
		Calmative, promotes rest.		Pillow	9
		Calmative, promotes rest.		Infusion is consumed.	9

Eremophila goodwinii F.Muell.	Unknown	Purgative	Leaves	Decoction is ingested.	9
Eremophila latrobei F.Muell.	Crimson turkey bush, native fuchsia, Latrobe's emu bush, grey fuchsia, warty fuchsia, Georgina poison bush (English), Mintjingka and Ngarankuta (Yankunytjatjara and Pitjantjatjara), Miyinypa (Warlpiri)	Body wash for scabies.	Leaves	Decoction, applied topically.	12
		Smoke is inhaled to treat general illness		Burned and inhaled.	15,18,19
		Colds and antibacterial agent for sore throat		Decoction is ingested.	3
		Malaise, colds and influenza		Decoction, applied topically.	3
Eremophila longifolia (R.Br.) F.Muell.	Berrigan, emu bush, dogwood, weeping emu bush (English), Otenerrenge (Aranda), Tulypurpa (Pitjantjatjara, Yankunytjatjara), Julpur (Pitjantjatjara)	Antiseptic for minor wounds, dermatological lesions, skin/body wash	Leaves	Decoction, applied topically.	1,20
		To enhance wellness of mothers and newborn babies.		Infusion. Application was not specified.	6,21,22
		Eye wash and antiseptic for ophtalmic complaints .		Decoction is used as an eyewash.	1,10
		Colds		Infusion is ingested.	23
		To relieve dermal irritation.		Decoction is applied topically.	23
Eremophila lucida Chinnock	Unknown	General wellness	Leaves	Not specified.	24
Eremophila maculata (Ker Gawl.) F.Muell.	Fuchsia bush, spotted emu bush, native fuchsia, wild fuchsia, spotted fuchsia, (English), Wedgerra (local dialect in the Hungerford district beyond the Darling River), Tchuldani (local dialect in the Cooper's Creek region near Lake Eyre)	To treat colds	Leaves	Poultice, preparation was not specified.	4,5,25

Eremophila mitchellii Benth.	Unknown	Used to treat respiratory conditions.	Twigs	Burned and the smoked is inhaled.	16
			Wood	Burned and the smoked is inhaled.	26
Eremophila neglecta J.M.Black	Unknown	General well-being.	Leaves	Infusion is ingested.	9
Eremophila paisley F.Muell.	Unknown	Used as a body wash to treat scabies.	Leaves and twigs	Infusion is used as body wash.	9,1,27
Eremophila sturtii R.Br.	Turpentine bush, narrow-leaved emu bush, small sandalwood, scented sandalwood, turpentine emu bush, budda bush/ budddha bush (English), Lpurta lpurta (Aranda), Watara (Yankunytjatjara)	Backaches	Branches	Preparation not specified. Applied topically.	20
			Whole shrub	Burnt ashes are rubbed onto the affected area.	8
		Fly repellant.	Leaves	Preparation and application were not specified.	3
		Wash for sores and cuts.		Decoction is used topically.	3
		Colds, flu, and sore eyes.		Hot bath containing leaves (infusion).	4,5,14
		Cough and respiratory infections.		Preparation and application were not specified.	4,5,1
		Used to treat diarrhoea.		Decoction is ingested.	14

Table S2. *Eremophila* spp. that have been screened for therapeutic properties.

Species	Indication	Part used	Test method and/or results	References
E. alternifolia	Inhibition of bacterial growth	Leaf extract	Inhibited *B. cereus, S. aureus* and *S. pyogenes* growth. MIC values were not reported.	28
	Antimicrobial activity	Leaf extract	*Staphylococcus aureus* (anstibiotic sensitive and MRSA strains). MIC values were not reported.	29
	Inhibition of bacterial growth	Leaf extract	Inhibits *Listeria monocytogenes* growth in full cream milk, skim milk, salami, pâté and cheese.	30
	Antifungal activity	Leaf extract	*Cryptococcus gattii* and *Cryptococcus neoformans*: compound 8,19-dihydroxyserrulat-14-ene inhibited *C. albicans, C. krusei* and *C. glabrata* growth, with MIC values between 8 and 512 μg/mL.	31
	Antibacterial activity	Leaf extract	Inhibit macromolecular biosynthetic pathways and compromise cell membrane integrity of Gram-positive bacteria.	31
E. bignoniiflora	Antibacterial activity	Leaf essential oil	The essential oil displayed noteworthy antibacterial activity against a panel of bacteria including *Staphylococcus epidermidis, K. aerogenes, P. aeruginosa* in agar diffusion assays. MIC values were not determined.	32
	Antifungal activity and ringworm	Leaf essential oil	Weak inhibitory activity against ringworm-causing *Trichophyton* spp. MIC values were not determined.	32
	Anti-candiadiasis activity	Leaf essential oil	Inhibitory activity against *C. albicans*, although MIC values were not determined.	32
	Anti-diabetic activity	Leaf extract	Protein tyrosine phosphatase 1B (PTP1B) inhibitory activity (IC_{50} = 23.9 μg/mL). (5H)-Furanone sesquiterpenoids exhibited moderate PTP1B inhibitory activity (IC_{50} values between 41.4 and 154.5 μM.	33
E. duttonii	Inhibition of bacterial growth	Leaf extract	Inhibits *Listeria monocytogenes* growth in full cream milk, skim milk, salami, pâté and cheese.	30

	Inhibition of bacterial growth	Leaf extract	Weak to moderate inhibition of *B. cereus, E. faecalis, S. aureus* and *S. pyogenes* in agar diffusion assays. MIC values were not determined.	28
	Antibacterial activity	Leaf extract	Noteworthy inhibition of *Clostridium perfringens, Clostridium sporogenes* and *Listeria monocytogenes* in a agar diffusion assay, with zones of inhibition 12-15 mm. MIC values were not determined.	34
E. freelingii	Inhibition of bacterial growth	Leaf extract	Weak to moderate inhibition of *B. cereus* in agar diffusion assays. MIC values were not determined.	28
E. latrobei	Inhibition of bacterial growth	Leaf extract	Weak to moderate inhibition of *B. cereus* in agar diffusion assays. MIC values were not determined.	28
E. longifolia	Antiviral activity	Leaf extract	Interferes with a step in the replication cycle of Ross River virus, but was ineffective against poliovirus and human cytomegalovirus.	35
	Antibacterial activity	Leaf essential oil	Inhibitory activity against *Staphylococcus aureus, Staphylococcus epidermidis, Salmonella typhimium, Klebsiella aerogenes, Eschirechia coli, Streptococcus pneumonia* and *Bacillus cereus*, with MIC values of generally below 2% of the oil.	36
	Cardiac activities	Leaf extract	Verbascoside and geniposidic acid isolated from the extract inhibited negative chronotropism, negative inotropism, and coronary perfusion rates. IC_{50} values were not reported.	37
E. lucida	Anti-candiadiasis activity	Leaf essential oil	Inhibitory activity against *C. albicans*, with MIC values of approximately 0.1-2% of the oil.	36
E. maculata	Antibacterial activity	Leaves and resin	The sesquiterpenoid farnesal was identified and tested for antibacterial activity, with noteworthy inhibition of several *Staphylococcus* and *Streptococcus* species (MICs = 65 µg/mL).	29
	Antibacterial activity	Essential oil produced from aerial parts	Tested using a broth dilution method. Low to moderate inhibitory activity against multiple *Staphylococcus* (including several MRSA strains) and *Streptococcus species* (MICs between 500-4000 µg/mL; MBCs 1000-4000 µg/mL).	38

E. neglecta	Anti-inflammatory effects	Serrulatane diterpenoids isolated from leaf extracts	Significant inhibitory effect on tumor necrosis factor TNF-α and IL-6 from BMDM cells.	39
	Biofilm removal		Significantly inhibited *Staphylococcus aureus* and *Staphylococcus epidermidis* biofilm formation.	39
	Antibacterial activity	Serrulatane diterpenoids isolated from leaf extracts	Significantly inhibited *Staphylococcus aureus* and *Staphylococcus epidermidis* biofilm formation (MICs = 3-25 μg/mL; MBCs = 6-316 μg/mL).	40
E. sturtii	Anti-inflammatory activity	Serrulatane diterpenoids isolated from fresh leaves	Serrulatic acid inhibited COX-1 and COX-2 by 99% and 97% respectively at 1 mg/mL, but was completely ineffective against 5-LOX.	41
	Inhibition of bacterial growth	Leaf extract	Weak to moderate inhibition of *B. cereus* in agar diffusion assays. MIC values were not determined.	28
	Antimicrobial activity	Fresh leaves	*Staphylococcus aureus* MIC = 1.0 mg/ml (ethanolic extract), MIC = 0.25 mg/mL (ethyl acetate fraction), MBC = 200 mg/mL; (3,8-dihydroxyserrulatic acid) MBC = 15 mg/mL (serrulatic acid).	41
Eremophila serrulata (A.Cunn. ex A.DC.) Druce	Antibacterial activity	Leaves	*Staphylococcus aureus* MICs = 16 to 250 mg/mL (9-methyl-3-(4-methyl-3-pentenyl)-2,3-dihydronaphtho[1,8-bc] pyran-7,8-dione and 8,20-diacetoxyserrulat-14-en-19-oic acid); MBC = 125 mg/mL (8,20-diacetoxyserrulat-14-en-19-oic acid).	42

References

1. Singab, A.N., Youssef, F.S., Ashour, M.L., Wink, M. The genus *Eremophila* (Scrophulariaceae): an ethnobotanical, biological and phytochemical review. *J Pharm Pharmacol* **2013**, 65(9), 1239-1279.
2. Tindale, N.B. Vocabulary of Pitjandjarra, the language of the native of the Great Western Desert, South Australian Museum Collection **1931**.
3. Smith, N.M. Ethnobotanical field notes from the Northern Territory, Australia. *J Adel Bot Gard* **1991**, 14(1), 1-65.
4. Cock, I.E. Medicinal and aromatic plants–Australia. Ethnopharmacology, Encyclopedia of Life Support Systems (EOLSS), **2011**. Available at http://www.eolss.net/Sample-Chapters/C03/E6-79-73.pdf
5. Lassak, E.V., McCarthy, T. Australian Medicinal Plants, A complete guide to identification and usage. Reed New Holland Publishers, Australia **2011**.
6. Richmond, G.S. A review of the use of *Eremophila* (Myoporaceae) by Australian Aborigines. *J Adel Bot Gard* **1993**, 101-107.
7. Barr, A. Traditional Bush Medicines: An Aboriginal Pharmacopoeia. Aboriginal Communities of the Northern Territory of Australia. **1988**; Greenhouse Publications, Richmond, Australia.
8. Bowen, S.E. Taxonomic studies in the Myoporaceae. BSc. (Hons) Thesis, The University of New England **1975**.
9. Latz, P.K. Bushfires and Bushtucker. MA Thesis. The University of New England **1982**.
10. O'Connell, J.F., Latz, P.K., Barnett, P. Traditional and modem plant use among the Alyawara of Central Australia. *Econ Bot* **1983**, 31, 80- 109.
11. Reid, E.J., Betts, T.J. Records of Western Australian plants used by aboriginals as medicinal agents. *Planta Med* **1979**, 36, 164-173.
12. Cleland, J.B., Johnston, T.H. The native names and uses of plants at Haasts Bluff, Central Australia. *Trans Proc R Soc S Aus* **1959**, 82, 123-140.
13. Maconochie, J.R. Pitjantjara names of some central Australian plants. *S Aus Nat* **1970**, 44(4), 75-77.
14. Barr, A. Traditional Bush Medicines: An Aboriginal Pharmacopoeia. Aboriginal Communities of the Northern Territory of Australia. **1988**; Greenhouse Publications, Richmond, Australia.
15. Meggitt, M.J. Desert people: a study of the Walbiri aborigines of Central Australia. Angus and Robertson Publications, Australia **1962**.
16. Low, T. Bush medicine: A pharmacopoeia of natural remedies. Collins/Angus and Robertson Publications, Australia **1990**.
17. Richmond, G.S., Ghisalberti, E.L. The Australian desert shrub *Eremophila* (Myoporaceae): Medicinal, cultural, horticultural and phytochemical uses. *Econ Bot* **1994**, 48(1), 35-59.
18. Birch, A.J., Massy-Westropp, R., Wright, S.E., Matsuura, T., Sutherland, M.D. lpomearone and ngaione. *Chem Ind* **1954**, 902.
19. Hegarty, B.F., Kelly, J.F., Park, R.J., Sutherland, M.D. Terpenoid chemistry XVII. (-)-Ngaione. a toxic constituent of *Myoporum desertii*. The absolute configuration of (-)-ngaione. *Aus J Chem* **1970**, 23, 107-117.
20. Silberbauer, G.B. Ecology of the Ernabella aboriginal community. *Anthropol Forum* **1971**, 3(1), 21-36.
21. Cleland, J.B., Johnston, T.H. The ecology of the aborigines of Central Australia. *Trans Proc R Soc S Aus* **1933**, 57, 113-124.
22. Cleland, J.B., Johnston, T.H. Notes on native names and uses of plants in the Musgrave Ranges region. *Oceania* **1937**, 8(2), 208-215.
23. Spencer, W.B., Gillen, F.J. The native tribes of Central Australia. Dover Publications **1969**, New York, USA.
24. Biva, I.J., Ndi, C.P., Griesser, H.J., Semple, S.J. Antibacterial constituents of *Eremophila alternifolia*: An Australian aboriginal traditional medicinal plant. *J Ethnopharmacol* **2016**, 182, 1-9.
25. Cunningham, G.M., Mueller, W.E., Milthorpe, P.L., Leigh, J.H. Plants of western New South Wales. NSW Government Printing Office **1981**.
26. Cribb, A.B., Crib, J.W. Useful wild plants in Australia. Collins Publications, Sydney, **1982**.
27. Ghisalberti, E.L. The phytochemistry of the Myoporaceae. *Phytochem* **1994**, 35, 7-33.
28. Palombo, E.A., Semple, S.J. Antibacterial activity of traditional Australian medicinal plants. *J Ethnopharmacol* **2001**, 77(2-3), 151-157.

29. Biva, I.J., Ndi, C.P., Semple, S.J., Griesser, H.J. Antibacterial performance of terpenoids from the Australian plant *Eremophila lucida*. *Antibiotics* **2019**, 8(2), 63.
30. Owen, R.J., Palombo, E.A. Anti-listerial activity of ethanolic extracts of medicinal plants, *Eremophila alternifolia* and *Eremophila duttonii*, in food homogenates and milk. *Food Control* **2007**, 18(5), 387-390.
31. Hossain, M.A., Biva, I.J., Kidd, S.E., Whittle, J.D., Griesser, H.J., Coad, B.R. Antifungal activity in compounds from the Australian desert plant *Eremophila alternifolia* with potency against *Cryptococcus* spp. *Antibiotics* **2019**, 8(2), 34.
32. Sandgrove, N.J., Hitchcock, M., Watson, K., Jones, G.L. Chemical and biological characterization of novel essential oils from *Eremophila bignoniiflora* (F. Muell) (Myoporaceae): A traditional Aboriginal Australian bush medicine. *Phytother Res* **2013**, 27(10), 1508-1516.
33. Zhao, Y., Kjaerulff, L., Kongstad, K.T., Heskes, A.M., Moller, B.L., Staerk, D. 2 (5H)-Furanone sesquiterpenes from *Eremophila bignoniiflora*: High-resolution inhibition profiling and PTP1B inhibitory activity. *Phytochem* **2019**, 166, 112054.
34. Shah, A., Cross, R.F., Palombo, E.A. Identification of the antibacterial component of an ethanolic extract of the Australian medicinal plant, *Eremophila duttonii*. *Phytother Res* **2004**, 18(8), 615-618.
35. Semple, S.J., Reynolds, G.D., O'Leary, M.C., Flower, R.L.P. Screening of Australian medicinal plants for antiviral activity. *J Ethnopharmacol* **1998**, 60(2), 163-172.
36. Sandgrove, N.J., Mijajlovic, S., Tucker, D.J., Watson, K., Jones, G.L. Characterization and bioactivity of essential oils from novel chemotypes of *Eremophila longifolia* (F. Muell) (Myoporaceae): a highly valued traditional Australian medicine. *Flavour Frag J* **2011**, 26(5), 341-350.
37. Pennacchio, M., Syah, Y.M., Ghisalberti, E.L., Alexander, E. Cardioactive compounds from *Eremophila* species. *J Ethnopharmacol* **1996**, 53(1), 21-27.
38. Youssef, F.S., Hamoud, R., Ashour, M.L., Singab, A.N., Wink, M. Volatile oils from the aerial parts of *Eremophila maculata* and their antimicrobial activity. *Chem Biodivers* **2014**, 11(5), 831-841.
39. Mon, H.H., Christo, S.N., Ndi, C.P., Jasieniak, M., Rickard, H., Hayball, J.D., Griesser, H.J., Semple, S.J. Serrulatane diterpenoid from *Eremophila neglecta* exhibits bacterial biofilm dispersion and inhibits release of pro-inflammatory cytokines from activated macrophages. *J Nat Prod* **2015**, 78(12), 3031-3040.
40. Anakok, O.F., Ndi, C.P., Barton, M.D., Griesser, H.J., Semple, S.J. Antibacterial spectrum and cytotoxic activities of serrulatane compounds from the Australian medicinal plant *Eremophila neglecta*. *J Appl Microbiol* **2012**, 112(1), 197-204.
41. Liu, Q., Harrington, D., Kohen, J.L., Vermulpad, S., Jamie, J.F. Bactericidal and cyclooxygenase inhibitory diterpenes from *Eremophila sturtii*. *Phytochem* **2006**, 67(12), 1256-1261.
42. Ndi, C.P., Semple, S.J., Griesser, H.J., Pyke, S.M., Barton, M.D. Antimicrobial compounds from *Eremophila serrulata*. *Phytochem* **2007**, 68(21), 2684-2690.
